# Residents’ Support for Tourism Amidst the COVID-19 Era: An Application of Social Amplification of Risk Framework and Knowledge, Attitudes, and Practices Theory

**DOI:** 10.3390/ijerph19063736

**Published:** 2022-03-21

**Authors:** Ke Shen, Jian Yang

**Affiliations:** 1 School of Tourism, Huangshan University, Huangshan 245041, China; jordan.shen@hsu.edu.cn; 2 School of Journalism and Communication, Guangzhou University, Guangzhou 510006, China

**Keywords:** residents’ support for tourism, SARF, KAP theory, risk perception

## Abstract

Given that the concept of risk perception stems primarily from consumer behaviour, tourism research has tended to address the issue from tourists’ perspective, resulting in a lack of consideration of destination residents’ risk perception and its impact on their attitudes and subsequent behaviour. Based on the social amplification of risk framework (SARF) and the knowledge, attitudes, and practices (KAP) theory, this study constructed a theoretical model to deepen the understanding of destination residents’ support for tourism. Results indicate that residents’ social media use, knowledge of COVID-19 and attitudes to tourism and tourists are all positively related to their support for tourism. Furthermore, residents’ risk perception is negatively associated with their attitudes to tourism, attitudes to tourists and support for tourism. However, the relationship between residents’ social media use and risk perception was not confirmed. Theoretical and managerial implications were discussed.

## 1. Introduction

The COVID-19 outbreak was announced toward the end of 2019 in some parts of the world and spread to the entire globe in 2020, causing travel restrictions at home and in many countries. These control measures have hit the tourism industry the hardest [1]. The real concern is that the tourism industry will most likely return to its development status quo pre-COVID-19 in at least 2 to 5 years [2]. Thus, a plethora of studies have been conducted to examine what impact COVID-19 has had on the travel industry and how the travel industry survives the COVID-19 [3,4]. Among the studies, the research on risk perception (RP) has received considerable attention [1,5,6,7].

The COVID-19 pandemic was quickly contained in some countries (e.g., China) after strict prevention and control measures were implemented, resulting in the rapid recovery of domestic tourism [8]. However, the revival of domestic tourism has left residents of tourist destinations in a precarious position. In many cases, avoiding COVID-19 hotspots would be comparatively easy for tourists, switching their plans if their destination turned out to be a danger zone. However, for residents, the options are often limited [9]. Given the prolonged incubation period and the asymptomatic nature of COVID-19, residents may have difficulty identifying and avoiding infectious tourists, which leads them to seclude themselves or accept risky tourists [1]. As an essential step to ensure a destination’s tourism sustainability, the role of residents’ support for tourism (SUPT) has been demonstrated in many studies [1,7,10,11]. In the face of the spread of COVID-19 and sporadic outbreaks in some areas, residents’ SUPT has become especially important but has also brought them risk. As a matter of fact, the resistance and hostility of residents towards tourists amidst the COVID-19 era demonstrate a real and substantial risk perception among residents [1]. Thus, research on residents’ RP and its impact on their subsequent attitudes and SUPT amidst the COVID-19 era are urgent and beneficial. Nevertheless, given that the concept of RP stems primarily from consumer behaviour, tourism research has tended to address the issue from tourists’ perspective [12], For instance, the concept of tourism risk perception is often conceptualized as tourists’ subjective feelings, objective evaluation and cognitive of exceeding the threshold portion of the negative consequences [13], and most studies are focused on the impact of tourists’ risk perception on their attitudes and behavioural intention [14,15,16,17]. However, residents’ perspectives of risk perception are absent from the literature [7,18]. In such a case, the social amplification of risk framework (SARF) and knowledge, attitudes, and practices (KAP) theory may be used to investigate the gap in the literature.

As one of the most comprehensive frameworks for risk research [19], the SARF is primarily concerned with illustrating how RP can be amplified or attenuated through the process of information communication [20]. However, ‘tourism research grounded in SARF has been limited in scope and is sparse’ (p. 449) [21]. The SARF’s original emphasis was on the role of traditional media (e.g., television, radio and newspapers) in previous literature, but its theoretical framework has been proven helpful in understanding how social media use (SMU) influences RP [22]. Although SMU has greatly changed how individuals seek and share information, research on these new sources of information is still scarce [23]. The gap in the literature makes this study an essential contribution to theory and practice.

As another theoretical foundation of this study, KAP theory remains one of the most frequently used models to explain how individuals’ knowledge and attitudes can influence their behaviour [24]. In the extant literature, it has been widely applied in the field of medicine [25]. However, its utilization in relation to tourism and travel is still in its infant stage [26]. Among the few studies grounded in KAP theory in tourism literature, the majority aims to understand tourists’ concerns regarding travel-related infectious diseases [27,28]. Nevertheless, to our knowledge, no research has attempted to analyse residents’ SUPT in light of KAP theory.

**Figure 1:**
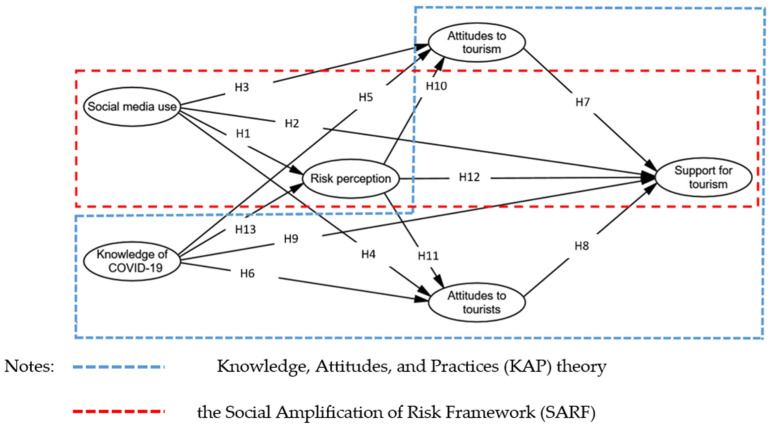
Theoretical model.

Thus, grounded in the SARF and KAP theory, this study constructs an integrated theoretical model to examine residents’ SUPT. Furthermore, given that limited research has investigated residents’ attitudes to tourism (ATT) and attitudes to tourists (ATTT) simultaneously to understand their SUPT [10], this study divides residents’ attitudes into two types of categories (i.e., ATT and ATTT) based on differential objects and explains their respective impact on residents’ SUPT (Figure 1).

## 2. Literature Review

### 2.1. The Social Amplification of Risk Framework (SARF)

Rooted in communication theory, the SARF mainly depicts that individuals’ discernment of risk will be amplified or attenuated when risk information is transferred, and their subsequent behaviour will also be impacted [20]. During the transfer of risk information, media serves as amplification or attenuation stations to form individuals’ awareness of events [29]. In the extant literature, the SARF was primarily concerned with the role of traditional media during the pre-internet period [22,23]. As the internet and social media have become more prevalent, this new way of seeking and sharing information has dramatically changed people’s life. Compared with traditional media, social media works in different ways to influence public opinion [30]. However, research on social media as amplification or attenuation stations and its influence on individuals’ behavioural reactions amidst the COVID-19 era remains scarce [22]. As this study focuses on the reactions of message receivers (i.e., residents) to risk information (i.e., COVID-19 information) delivered through amplification or attenuation stations (i.e., social media), SARF theory is considered to be a suitable theoretical framework [23].

A growing number of people are seeking and sharing health-related information via social media [31], which has revolutionized tourism [32]. However, tourism studies grounded in the SARF are largely ignored in the existing literature [21], and most of them have adopted qualitative approaches [33], with Cahyanto and Liu-Lastres [21] and Shakeela and Becken [33] as exceptions. Thus, this study adopts a quantitative approach to understand residents’ SUPT from a SARF perspective.

Guided by the SARF, Cahyanto and Liu-Lastres [21] found that media exposure was related to visitors’ risk perception of Florida’s Red Tide outbreak and their behavioural responses. With an investigation in the extremely low-lying Maldives, Shakeela and Becken [33] found that climate change risks were amplified via international media but attenuated through national media for international audiences. Furthermore, a survey of 679 US citizens revealed that their social media interaction about COVID-19 was significantly related to their risk perception of COVID-19 [22]. In Wuhan City (China), Zhong et al. [34] reported that social media usage among residents was positively related to their health behaviour change. An investigation of 550 Chinese citizens has confirmed the relationship between media exposure and their pro-environmental behaviour [35]. On the basis of the SARF and the discussion above, the following hypotheses were proposed:

**Hypothesis** **1.** **(H1).** *Residents’ SMU is associated with their RP.*

**Hypothesis** **2.** **(H2).** *Residents’ SMU is associated with their SUPT.*

In the SARF, social media use can affect individuals’ risk perception and subsequent behaviour, and some studies have shown that it impacts individual attitudes. Kane et al. [36] indicated that Facebook, as one example of a social media channel, may not directly lead to a change in travel behaviour but may influence attitudes and values that are likely to result in changes in travel behaviour over time. However, most research focusing on social media in tourism comes from a marketing perspective, concentrating primarily on its influence on travellers’ decision-making process [37]. Thus, few studies have touched on this issue from a residents’ perspective, with Lu et al. [38] and Nunkoo, Gursoy and Dwivedi [30] as exceptions. By integrating the influence of presumed influence model, the elaboration likelihood model and social exchange theory, Nunkoo, Gursoy and Dwivedi [30] developed a research model and hypothesized the influence of social media on residents’ attitudes towards tourism, although data collection and testing are still required to confirm the hypothesis. Some other empirical evidence supports a similar hypothesis. With a sample of 505 residents in China and 449 residents in the US, Lu, Mihalik, Heere, Meng and Fairchild [38] confirmed that media content was related to their attitudes towards the Olympic bid. Furthermore, Yoo et al. [39] confirmed the significant relationship between social media and college students’ smoking attitudes. Thus, the following hypotheses were posited:

**Hypothesis** **3.** **(H3).** *Residents’ SMU is associated with their ATT.*

**Hypothesis** **4.** **(H4).** *Residents’ SMU is associated with their ATTT.*

### 2.2. Knowledge, Attitudes and Practices (KAP) Theory

Developed by Mayo in the 1960s [24], KAP theory tells how communication increases knowledge, changes attitudes and improves behaviours by intervening in cognitive, affective and behavioural elements [40]. It is mainly comprised of three continuous processes: an individual’s understanding of healthy knowledge, the establishment of beliefs and attitudes and the adoption of subsequent health behaviours [24]. Knowledge underpins behaviour change, and attitudes motivate that change [24].

Considering that KAP theory is one of the core foundations for changing the behaviour of individuals concerning their health, most studies based on this model have been focused on the field of medicine [24,41,42,43]. Thus, studies grounded in KAP theory remain rare in the tourism literature [26] and residents’ support for tourism. Among the few tourism studies based on KAP theory, most were conducted to understand tourists’ knowledge of the disease, attitudes and disease-prevention behaviours during their journey [28,44,45]. An investigation in Taiwan revealed that air travellers with different types of trip purposes and occupational groups had significantly different knowledge, attitudes and behaviour related to influenza A [45]. Another similar study confirmed insufficient awareness of vaccine-preventable diseases, food safety and measures to prevent insect bites among air travellers in Muscat International Airport in Oman [44]. Some other types of tourist behaviour have been examined based on KAP theory, including responsible behaviour intention [25] and pro-environmental behavioural intention [46]. In China, Chen, Dai, Liu, Liu and Jia [25] found that tourists’ knowledge of travel risk was positively related to their behavioural attitude and responsible behavioural intention. Similarly, a survey in Huangshan National Park (China) has identified the significant role of tourists’ knowledge of environmental theory and environmental practice in determining their attitude toward behaviour, and the substantial role in attitude towards behaviour in determining their behavioural intention to bring litter down the Huangshan Mountain [46]. With a sample of 625 physicians in Hubei, China, Liu, Liu, Wang and Zhang [42] found that knowledge was positively associated with their attitude and behavioural intention to contain antibiotic prescriptions. On the basis of KAP theory and the above discussion, the following hypotheses were formulated:

**Hypothesis** **5.** **(H5).** *Residents’ KN is associated with their ATT.*

**Hypothesis** **6.** **(H6).** *Residents’ KN is associated with their ATTT.*

**Hypothesis** **7.** **(H7).** *Residents’ ATT is associated with their SUPT.*

**Hypothesis** **8.** **(H8).** *Residents’ ATTT is associated with their SUPT.*

**Hypothesis** **9.** **(H9).** *Residents’ KN is associated with their SUPT.*

### 2.3. Risk Perception

The term ‘risk perception’ refers to the way individuals think, feel and assess the uncertainty and negative outcomes of their decisions [1]. Travelling to places outside one’s usual residence implies uncertainty and risk, which makes tourism and risk intrinsically connected [47]. Thus, risk has become an important topic in recent literature since it was first introduced into tourism research in the 1990s [48]. Previous studies on risk were mainly focused on crisis events, such as the 9/11 attack [49] and North Korea’s nuclear tests [50]. Recent research has mainly focused on examining the influence of RP on tourists’ future behavioural intentions [17]. Nevertheless, given that the concept of RP stems primarily from consumer behaviour, tourism research has tended to address the issue from tourists’ perspective [12], resulting in a considerable lack of consideration of residents’ RP [1,7,18].

China has now largely controlled the COVID-19 epidemic, but sporadic outbreaks and the recovery of domestic tourism still put residents of tourist destinations in a dangerous situation, making studies on residents’ risk perception of COVID-19 urgent and significant. Numerous studies have demonstrated the relationship between tourists’ risk perception, attitudes and subsequent behaviours. For instance, a study by Choi et al. [51] revealed that consumers’ perceived risks negatively affected their attitude towards street food and their behavioural intention. Similarly, a survey conducted in South Korea found that tourists’ attitudes and behaviour intention to ‘untact’ tourism were strongly impacted by their perceptions of risk [52]. Rather [53] found that tourists’ risk perception of COVID-19 has a significant negative impact on their attitudes to travelling. To date, some studies have been conducted to confirm the relationship among residents’ risk perception of COVID-19, attitudes and their support for tourism. An investigation in Jeju Island has verified that residents’ risk perception was negatively associated with their support for tourism [1]. However, in another research in Georgia (US), Woosnam, Russell, Ribeiro, Denley, Rojas, Hadjidakis, Barr and Mower [7] found that residents’ risk perception of COVID-19 was not a significant predictor of their pro-tourism behaviour, despite confirmation of the significant relationship between risk perception and attitudes. These inconsistent findings make more research necessary to confirm the relationship between residents’ risk perception of COVID-19 and their support for tourism. Given these findings, the following hypotheses were postulated:

**Hypothesis** **10.** **(H10).** *Residents’ RP is associated with their ATT.*

**Hypothesis** **11.** **(H11).** *Residents’ RP is associated with their ATTT.*

**Hypothesis** **12.** **(H12).** *Residents’ RP is associated with their SUPT.*

As a consequence of the enormous influence of risk perception on individuals’ attitudes and behaviours, many studies have been dedicated to exploring the factors that influence risk perception [9,14,54]. In general, the influencing factors of perceived risk can be divided into two groups. The first one pertains to individuals’ trust in tourism providers, healthcare institutions, governmental officials and knowledge about the risk, whereas the second one pertains to individuals’ personality traits [55]. Given that tourism research has tended to understand risk perception from a tourists’ perspective [7], the majority of existing research on the influencing factors of risk perception were conducted from a tourists’ perspective. However, existing studies have noted some inconsistencies regarding the relationship between risk perception and knowledge. In Nigeria, Iorfa et al. [56] reported that higher COVID-19 knowledge leads to a greater risk perception. Nevertheless, a study by Zhu and Deng [14] has verified the negative relationship between knowledge of pneumonia and risk perception. Given the inconsistency of these findings, more research is necessary to clarify the impact of knowledge on risk perception. Thus, we hypothesized the following:

**Hypothesis** **13.** **(H13).** *Residents’ KN is associated with their RP.*

## 3. Methodology

### 3.1. Instrument Design and Measurements

The survey instrument was divided into two sections. The six constructs in the research model were represented by 26 items adapted from existing studies in Section 1. Residents’ support for tourism (four items) was adapted from Joo, Xu, Lee, Lee and Woosnam [1]. A sample item states: “I believe Huangshan tourism should be actively promoted during the pandemic.” Residents’ attitudes to tourism (four items) and attitudes to tourists (four items) were adapted from Shen, Yang and Geng [10]. Sample items read as follows: “I believe tourism is a good activity for Huangshan” and “For me, the tourists who visit Huangshan is positive.” Residents’ risk perception of COVID-19 (four items) was adapted from Woosnam, Russell, Ribeiro, Denley, Rojas, Hadjidakis, Barr and Mower [7]. A sample item states: “Incoming tourists increase the risk of COVID-19 infection.” Residents’ social media use was assessed based on how often residents consumed COVID-19 news from five of the most popular Chinese social media platforms [57]. A sample item read as follows: “Over the past week, how often have you consumed COVID-19 news from WeChat?” Residents’ knowledge of COVID-19 (five items) was adapted from Zhu and Deng [14]. A sample item states: “I know about the initial cause of COVID-19.” To assess each item, we used a five-point Likert scale from ‘strongly disagree’ to ‘strongly agree’, except for social media use, which was rated from ‘never’ to ‘always’. In Section 2, the five demographic questions included gender, marriage, age, education and income. Detailed information about our measurement items can be found in Appendix A.

### 3.2. Study Area and Data Collection

**Figure 2:**
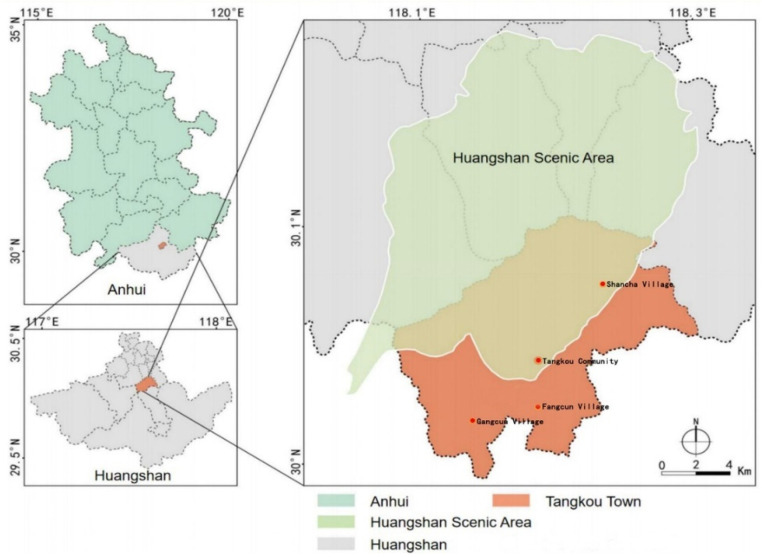
Location of Tangkou Community, Gangcun Village, Fangcun Village and Shancha Village. Source: Modified from Wu et al. [58].

Huangshan Scenic Area (HSA) is situated in the southernmost area of Anhui province and is considered one of the top ten scenic spots in China. As a typical mountain scenic spot, HSA was proclaimed a World Heritage Site in 1989 for its distinctive natural scenery and splendid cultural heritage. HSA is surrounded by several towns, among which Tangkou Town has become the largest tourist reception and distribution centre by its unique geographical location, convenient transportation and well-designed cable car system throughout the scenic area. To date, the area has 28 travel agencies and over 540 catering and accommodation establishments, with more than 19,000 beds in town. COVID-19 has brought a heavy hit to the tourism industry in HSA, but with strong control measures and large-scale vaccination of COVID-19, many domestic tourists choose to enter HSA from Tangkou Town, resulting in a phenomenal domestic tourism increase in HSA. Thus, Tangkou Town was selected as the study area for this study. Tangkou Town consists of three villages and one community: Tangkou Community, Gangcun Village, Fangcun Village and Shancha Village (Figure 2). To maximize the sample’s representativeness and minimize any potential spatial biases, we surveyed all four villages and communities and sized the sample according to the number of households within each village or community (Table 1).

A convenience sampling method was used for data collection in each village and community from 10 to 26 November 2021. The questionnaires were solicited face to face with the assistance of a local government agent. A total of 400 questionnaires have been distributed, and 392 residents agreed to complete them. All questionnaires are distributed and collected on-site. In case of missing items, we checked the questionnaires immediately after collection. The data for the final analysis consisted of 382 valid questionnaires after eliminating 10 questionnaires that were filled out randomly. According to an a priori sample size calculation suggested by Gursoy et al. [59], the recommended minimum sample size is 161 (anticipated effect size = 0.3; desired statistical power level = 0.8; number of latent variables = 6; number of observed variables = 26; probability level = 0.05), thus 382 is sufficient for this study.

### 3.3. Common Method Bias

Given that all the questionnaires were collected from the same source, common method bias (CMB) needed to be assessed. Suggested by Kock [60], a full collinearity assessment approach was employed to diagnose CMB. Results from the full collinearity test indicated that the research model in this study was free of CMB because all variance inflation factors were less than 3.3.

### 3.4. Partial Least Squares Structural Equation Modelling

Partial least squares structural equation modelling (PLS-SEM) is superior to covariance-based structural equation modelling (CB-SEM) in terms of sample size and data normality and for complex models [61]. Thus, this study chose PLS-SEM to analyse the research model. SPSS 25 (IBM. Almonk, New York, U.S.A) and SmartPLS 3.3.2 (SmartPLS GmbH. Gewerbering, Oststeinbek, Germany) were used to test the research model following the two-step method suggested by Hair Jr, Hult, Ringle and Sarstedt [61]: the measurement model and the structural model.

## 4. Results

### 4.1. Sampling Profile

In Table 2, the proportion of males and females among the respondents was equal, in line with the gender ratio among mainland Chinese residents. In the way of age, the group of 31–40 years accounts for more than one-third of the 382 respondents, while those 51 years or older make up the least portion (19.6%). In terms of education level, undergraduate accounted for the largest proportion (40.1%) followed by junior college (37.4%). Regarding personal monthly income, the proportions of highest-income (≧ CNY 8001) and lowest-income (≦ CNY 3000) groups are not very large, representing only 5.5% and 3.9% respectively, and the majority of respondents are in the middle-income range.

### 4.2. Measurement Model

In this stage, measurement model evaluation focused on reliability and validity. As Table 3 demonstrated, factor loadings (0.720–0.884), Cronbach’s alpha (0.720–0.884) and composite reliability (0.869–0.913) were all above the shortcut of 0.708, 0.7 and 0.7, respectively, indicating that the measurement model reliability is satisfactory [62]. Furthermore, the average variance extracted (0.624–0.712) of each construct are all greater than the cut-off of 0.5, supporting adequate convergent validity of the measurement model [62].

The discriminant validity of the measurement model was evaluated using two methods: Fornell–Larcker criterion analysis [63] and the heterotrait–monotrait ratio of correlations (HTMT) [64]. Table 4 displays the square root of each AVE as the diagonal (in bold), the correlation coefficient between the constructs below the diagonal and the HTMT value above the diagonal, which confirms the discriminant validity of the measurement model.

### 4.3. Structural Model

The coefficient of determination (R^2^), the blindfolding-based cross-validated redundancy measure Q^2,^ and the statistical significance and relevance of the path coefficients suggested by Hair, Risher, Sarstedt and Ringle [62] were employed to assess the structural model. The R^2^-values in this study (ATT: 0.131; RP: 0.115; ATTT: 0.128; SUPT: 0.370) were all greater than the minimum cut-off value suggested by Falk and Miller [65]. The Q^2^-values (ATT: 0.086; RP: 0.069; ATTT: 0.073; SUPT: 0.230) were all larger than zero, establishing acceptable predictive relevance [62].

The significance and relevance of the path coefficients were assessed using a resampling method (5000 resamples). Hypotheses 1, 2, 3 and 4 examined the role of residents’ SMU on their RP, ATT, ATTT and SUPT. As listed in Table 5, residents’ SMU had a significant and positive impact on their SUPT, ATT, and ATTT (β = 0.179, *p* < 0.001; β = 0.147, *p* = 0.006; β = 0.158, *p* = 0.008, respectively), thus supporting H2, H3 and H4. However, residents’ SMU did not significantly predict their RP (β = 0.03, *p* = 0.628). Thus, H1 was rejected. The relationships between residents’ KN and their ATT, ATTT, SUPT and RP were tested through hypotheses 5, 6, 9 and 13. Residents’ KN significantly impacted their ATT, ATTT, SUPT and RP (β = 0.309, *p* < 0.001; β = 0.304, *p* < 0.001; β = 0.113, *p* = 0.036; β = 0.330, *p* < 0.001, respectively), as predicted by H5, H6, H9 and H13, respectively. Hypotheses 7 and 8 looked at the impact that residents’ ATT and ATTT had on their SUPT, which was confirmed by standardized path coefficients from ATT (β = 0.321, *p* < 0.001) and ATTT (β = 0.211, *p* < 0.001) on SUPT being significant and positive, thus supporting H7 and H8. In other words, residents with more positive attitudes were more inclined to support local tourism development. Finally, the role of residents’ RP was examined concerning their ATT, ATTT and SUPT through hypotheses 10, 11 and 12. Residents’ RP was found to relate to their ATT (β = −0.185, *p* = 0.002), ATTT (β = −0.113, *p* = 0.037), and SUPT (β = −0.164, *p* = 0.001) negatively and significantly, thus respectively confirming H10, H11 and H12, indicating that greater perceived risk leads to negative attitudes and lower support for tourism.

Residents’ ATT, RP and ATTT are suggested to serve as the mediator between SMU and SUPT, as well as KN and SUPT, which was tested using a resampling method (5000 resamples). As displayed in Table 6, all the mediation effects have been confirmed except for the indirect impact of SMU on SUPT through RP.

## 5. Discussion and Conclusions

### 5.1. Discussion

Based on the SARF and KAP theory, this study constructed a theoretical model to deepen the understanding of destination residents’ SUPT amidst the COVID-19 era. Of the 13 hypothesized relationships, 12 were supported. These findings may contribute to the literature on residents’ support for local tourism development and provide practitioners with valuable insights.

Residents’ social media use significantly predicted their support for tourism (H2), attitudes to tourism (H3) and attitudes to tourists (H4), echoing similar findings in the previous literature [32,34,35,38,39,66]. However, residents’ social media use has no significant correlation with their risk perception of COVID-19, which contradicted with what the SARF has contended and conflicted with the findings of some previous studies [21,22,23]. The lack of support for H1 may be explained by the fact that the SARF depicts risk that could be amplified or attenuated in the process of transferring risk information [20] and no clear-cut criteria could be applied to differentiate attenuation from amplification [19]. Previous studies have also confirmed that risk perception may be amplified or attenuated in the process of transmission. For instance, media exposure was found positively related to risk perception [21,23], whereas a negative relationship between social media interaction on COVID-19 and risk perception has also been reported in some studies [22]. On the other hand, the SARF contends that technical risk assessment differs significantly from individual risk assessment. Simply put, the general public perception of risk is lower than that of risk experts in the case of the same particular hazard [21].

Regarding the relationships among residents’ knowledge of COVID-19, attitudes, and support for tourism (supporting H5, H6, H7, H8 and H9), our findings align with previous studies. It has been found that with the increase of tourism knowledge, residents in the village of Ngada, Indonesia, were more inclined to hold positive attitudes towards tourism [67]. Similarly, Zhang et al. [68] indicated that tourism knowledge was an important factor in determining residents’ attitudes toward tourism. Moreover, with a sample of 300 local Malaysian residents, Chang et al. [69] revealed that their knowledge about tourism was positively related to their support for tourism development. These findings further confirm the applicability and validity of KAP theory in the context of tourism, specifically as it relates to residents’ support for tourism.

The negative relationships between residents’ risk perception of COVID-19 and their attitudes and the risk perception of COVID-19 and support for tourism (confirming H10, H11 and H12) are consistent with preceding studies [1,18]. These findings indicate that, when individuals perceive more risk, they will be more inclined to adopt protective behaviours. For instance, residents who lived in Jeju considered incoming tourists to be a source of risk, and this risk perception led to a lower level of support for tourism [1]. In Turkey, the risk perception of COVID-19 by the host community is an essential determinant of their intended hospitable behaviour. In other words, the more risk they perceive, the less inclined they are to be hospitable. In this study, destination residents perceive that the arrival of tourists may put them in a dangerous situation amidst the COVID-19 era, their attitudes toward tourism and tourists will be more negative, and the willingness to support tourism will be lower. However, some studies have reported different findings. In Georgia (US), residents’ perceived risk of COVID-19 was not associated with their pro-tourism behaviour [7]. The different findings may be attributed to different study areas and COVID-19 prevention policies adopted by the government. In this study, a tourist attraction in China was selected as the study area, and the Chinese government adopted a zero-tolerance policy for COVID-19. The study by Woosnam, Russell, Ribeiro, Denley, Rojas, Hadjidakis, Barr and Mower [7] was carried out in Georgia (U.S.), where the U.S. government adopts a natural herd immunity policy [70]. Given these differences, the public risk perceptions of COVID-19 also vary widely, resulting in differences in subsequent behaviours.

Lastly, our finding of a positive relationship between residents’ knowledge of COVID-19 and risk perception (supporting H13) mirrored previous studies [56,71]. In Nigeria, Iorfa, Ottu, Oguntayo, Ayandele, Kolawole, Gandi, Dangiwa and Olapegba [56] found that individual with higher COVID-19 knowledge was inclined to hold greater risk perception. Similarly, a survey of potential Australian tourists to Oman, Jordan, and the UAE revealed that tourists’ knowledge was related to their risk perception [71]. Cahyanto and Liu-Lastres [21] stated that the general public perception of risk is lower than that of risk experts in the case of the same particular hazard. That is, the more people learn about a particular hazard, the closer they become ‘experts’. As a result, their risk perception of the hazard will be greater than the general public’s, which may explain why residents’ knowledge of COVID-19 positively influenced their risk perception.

### 5.2. Conclusions

This study constructed an integrated theoretical model to examine residents’ support for tourism using data from 382 residents living in Tangkou Town. Based on the findings, some conclusions can be drawn. Firstly, this study verifies the application of the SARF and KAP theory in the context of tourism, specifically as it relates to residents’ support for local tourism development. Secondly, destination residents’ risk perception has a negative influence on their attitudes and behaviour in the same way that tourists do. In other words, positive attitudes and support for tourism development were more prevalent among residents with less risk perception. Lastly, residents’ attitudes to tourism have been found to be the strongest antecedent to predict their support for tourism, followed by attitudes to tourists, which is consistent with most extant studies and some theories.

## 6. Implication

### 6.1. Theoretical Implications

This study examined residents’ support for tourism development by integrating the SARF and the KAP theory by taking two kinds of attitudes into account, contributing to tourism literature. Firstly, the present study is one of the first attempts to examine the impact of risk perception on individuals’ attitudes and subsequent behaviour from a resident perspective. In previous tourism literature, risk perception was mainly utilized to understand its influence on tourists’ behavioural intention. Nevertheless, COVID-19 has made residents’ risk perceptions particularly significant because it partially determines their willingness to support local tourism development and the sustainable development of destinations. Furthermore, the prolonged COVID-19 pandemic and the potential outbreak of other hazards such as COVID-19 posit a high chance of studies on residents’ risk perception to occupy an imperative position [1]. In this sense, this study contributes to the growing literature on risk perception and deepens a better understanding of residents’ support for tourism.

Secondly, to our best knowledge, this study is the first to employ and incorporate the SARF and KAP theory to improve the understanding of residents’ support for tourism. Given that the SARF and KAP theory have been largely ignored in the previous tourism literature [21,26], this study greatly expands the application of both in the context of tourism, specifically as it relates to residents’ support for local tourism development. Furthermore, knowledge of COVID-19 was found related to risk perception, and 37% of variance in residents’ support for tourism has been explained. Thus, the logical combination of the SARF and KAP theory is confirmed. The conceptual framework was presented to be adopted directly or adapted in future studies on residents’ risk perception and demonstrating the importance of linking complementary theories to understand tourism behaviour from a resident perspective [72].

Thirdly, the SARF-based tourism studies are largely absent in the existing literature [21], and most of them have adopted a qualitative research approach [33], which cannot quantify the impact [73]. Thus, this study developed a theoretical model based on the SARF and KAP theory, utilizing a quantitative research approach to understand residents’ support for tourism, which can extend the research scope and how the SARF can be applied. 

Lastly, among the five significant predictors of residents’ support for tourism, residents’ attitudes to tourism have the strongest effect, followed by residents’ attitudes to tourists, which was in line with previous studies [10,74,75]. These findings further confirmed the significant and positive relationship between attitudes and behaviour. Furthermore, two types of residents’ attitudes were introduced simultaneously into the theoretical model, filling the previous literature gap. On the one hand, attitudes to tourists were largely ignored [76]. On the other hand, rather than exploring and understanding the antecedents and outcomes of residents’ attitudes to tourists, the previous literature was largely descriptive, listing and describing their attitudes ‘about’ tourists [77].

### 6.2. Practical Implications

The findings of this study also provide some valuable insights for practitioners. First, our findings of negative impacts of residents’ risk perception of COVID-19 on their support for tourism indicate that some measures should be taken to reduce residents’ perception of risk to gain their support for tourism development and achieve sustainable development destinations. Specifically, destination management can implement a real-name reservation system for tourists. Only tourists who have made reservations and passed the qualification review can enter the scenic area. Qualifications to be reviewed include, but are not limited to, no travel history to high-risk regions, no history of close contact with confirmed cases and a negative nucleic acid test certificate. Furthermore, there may be benefits to limiting the total number of tourists that enter a destination at a given time, which will alleviate residents’ concerns and make tourists feel safer. Moreover, tourists entering destinations must wear masks and have their body temperature monitored, and contactless services are recommended if possible.

According to the findings, residents’ knowledge of COVID-19 is a significant factor influencing their support for tourism. Consequently, government agencies and destination management should disseminate correct knowledge among destination residents. For instance, they may benefit from holding certain knowledge contests on COVID-19 among the destination residents, and winners will be given certain rewards. Inviting some experts, such as physicians, into villages and communities to share knowledge and common misconceptions of COVID-19 is also important.

Social media use was found to influence residents’ support for tourism, which sheds new light on the importance of utilizing social media to enhance residents’ levels of support for tourism. For example, relevant departments and management should create official accounts on popular social media platforms in China (such as WeChat, Sina Weibo, Douyin and Toutiao). They should provide updated information every day on confirmed and suspected cases of COVID-19, especially their travel history and means of transportation.

Considering the significant effect of residents’ attitudes on their support for tourism, destination practitioners should foster favourable attitudes towards tourism and tourists among residents. For instance, a benefit-sharing system should be adopted, and more job opportunities should be provided to residents to ensure their legitimate rights and interests. Furthermore, residents should be allowed to participate in decision-making processes related to tourism planning, and even serve as the primary management personnel of the destination tourism corporate. Moreover, more measures should be undertaken to ensure that tourists behave in a civilized manner and do not interfere with residents’ daily life, which may generate favourable attitudes to tourists among residents.

## 7. Limitations and Future Studies

Despite its theoretical and practical significance, the study also has several limitations. Firstly, this study adopted a convenience sampling method to collect data in the four communities and villages, influencing the sample’s representativeness. Thus, future studies may adopt the probability sampling method to raise data representativeness. Secondly, our research team, assisted by a local government agent, went into residents’ houses to distribute and collect questionnaires during the day. In consequence, some residents who work outside during the daytime may not be chosen for the survey. Future studies should consider this data collection issue to improve the sample’s representativeness. Lastly, this study has only introduced SMU, KN, RP and attitudes into the model. Future studies may consider more constructs, such as tolerance [78] and crowding [79].

## Figures and Tables

**Table 1:** Sample distribution.

Village or Community	Household (N) ^a^	Distributed Sample	Returned Sample	Valid Sample	Invalid Sample
Gangcun Village	3602	130	127	125	2
Tangkou Community	2854	105	103	97	6
Fangcun Village	2414	85	82	82	0
Shancha Village	2342	80	80	78	2
Total	11,212	400	392	382	10

^a^ Data retrieved from http://www.tcmap.com.cn/anhui/huangshanqu_tangkouzhen.html (accessed on 4 November 2021).

**Table 2:** Results of measurement model.

Demographic	Categories	N (%)
Gender	Male	198 (51.8%)
	Female	184 (48.2%)
Marital status	Single	129 (33.8%)
	Married	246 (64.4%)
	Others	7 (1.8%)
Age	18–30	96 (25.1%)
	31–40	119 (31.2%)
	41–50	92 (24.1%)
	≧51	75 (19.6%)
Education	Middle school or less	55 (14.4%)
	Junior college	143 (37.4%)
	Undergraduate	153 (40.1%)
	Post-graduate or higher	31 (8.1%)
Personal monthly income	≦CNY 3000	15 (3.9%)
	CNY 3001–4000	45 (11.8%)
	CNY 4001–5000	93 (24.3%)
	CNY 5001–6000	104 (27.2%)
	CNY 6001–7000	62 (16.2%)
	CNY 7001–8000	42 (11.0%)
	≧CNY 8001	21 (5.5%)

**Table 3:** Results of measurement model.

Items	Factor Loading	Cronbach’s Alpha	Composite Reliability	Average Variance Extracted
Attitudes to tourists				
ATTT1	0.744	0.801	0.869	0.624
ATTT2	0.771			
ATTT3	0.866			
ATTT4	0.774			
Attitudes to tourism				
ATT1	0.835	0.866	0.908	0.712
ATT2	0.840			
ATT3	0.823			
ATT4	0.876			
Support for tourism				
SUPT1	0.809	0.822	0.883	0.655
SUPT2	0.720			
SUPT3	0.819			
SUPT4	0.882			
Knowledge of COVID-19				
KN1	0.745	0.882	0.913	0.679
KN2	0.863			
KN3	0.840			
KN4	0.868			
KN5	0.796			
Risk perception				
RP1	0.860	0.860	0.904	0.702
RP2	0.884			
RP3	0.824			
RP4	0.779			
Social media use				
SMU1	0.755	0.869	0.904	0.653
SMU2	0.859			
SMU3	0.826			
SMU4	0.780			
SMU5	0.815			

**Table 4:** Discriminant validity.

Constructs	ATT	ATTT	SUPT	KN	RP	SMU
ATT	**0.844**	0.667	0.596	0.325	0.094	0.228
ATTT	0.562	**0.790**	0.553	0.348	0.112	0.247
SUPT	0.520	0.466	**0.810**	0.299	0.162	0.345
KN	0.286	0.308	0.262	**0.824**	0.366	0.290
RP	−0.064	0.009	−0.123	0.338	**0.838**	0.138
SMU	0.208	0.226	0.304	0.267	0.118	**0.808**

Note: Bold fonts are the square root of the AVE.

**Table 5:** Results of structural model.

Hypotheses	Path	Original Sample	Standard Error	*t*-Values	*p*-Values	Support
H1	SMU → RP	0.030	0.063	0.484	0.628	NO
H2	SMU → SUPT	0.179	0.045	3.943	0.000	YES
H3	SMU → ATT	0.147	0.053	2.775	0.006	YES
H4	SMU → ATTT	0.158	0.060	2.649	0.008	YES
H5	KN → ATT	0.309	0.061	5.033	0.000	YES
H6	KN → ATTT	0.304	0.049	6.204	0.000	YES
H7	ATT → SUPT	0.321	0.059	5.469	0.000	YES
H8	ATTT → SUPT	0.211	0.051	4.131	0.000	YES
H9	KN → SUPT	0.113	0.054	2.101	0.036	YES
H10	RP → ATT	−0.185	0.059	3.166	0.002	YES
H11	RP → ATTT	−0.113	0.054	2.086	0.037	YES
H12	RP → SUPT	−0.164	0.049	3.336	0.001	YES
H13	KN → RP	0.330	0.066	5.022	0.000	YES

**Table 6:** Results of mediation effects.

Path	Original Sample	Standard Error	*t*-Value	*p*-Value
KN → ATT → SUPT	0.099	0.029	3.417	0.001
SMU → ATT → SUPT	0.047	0.021	2.299	0.022
KN → ATTT → SUPT	0.064	0.018	3.575	0.000
SMU → ATTT → SUPT	0.033	0.015	2.153	0.031
KN → RP → SUPT	−0.054	0.020	2.716	0.007
SMU → RP → SUPT	−0.005	0.011	0.451	0.652

## Data Availability

The data presented in this study are available on request from the corresponding author. The data are not publicly available due to privacy issues.

## Appendix A

Measurement Items

**Risk Perception of COVID-19**
Incoming tourists increase my anxiety/stress related to COVID-19 prevention.
Incoming tourists increase the risk of COVID-19 infection.
Incoming tourists increase inconvenience in outdoor activities.
Incoming tourists makes me reduce my outdoor activities.

**Support for Tourism**
I support making further investment to develop Huangshan tourism during the pandemic.
I support providing tourists with more effective services during the pandemic.
I support attracting more tourists to Huangshan during the pandemic.
I believe Huangshan tourism should be actively promoted during the pandemic.

**Knowledge of COVID-19**
I know about the initial cause of COVID-19.
I know about the harm caused by COVID-19.
I know about the length of incubation period of COVID-19.
I know about the current affected range of COVID-19.
I know about the preventive measures for COVID-19.

**Social Media Use**
Over the past week, how often have you consumed COVID-19 news from WeChat?
Over the past week, how often have you consumed COVID-19 news from Weibo?
Over the past week, how often have you consumed COVID-19 news from Douyin?
Over the past week, how often have you consumed COVID-19 news from QQ?
Over the past week, how often have you consumed COVID-19 news from Toutiao?

**Attitude to tourism**
I believe tourism generates positive benefits for Huangshan.
I believe tourism is a good activity for Huangshan.
I would like the tourism sector to continue to play a major role in Huangshan.
I believe tourism should be actively encouraged in Huangshan.

**Attitude to tourists**
For me, the tourists who visit Huangshan is pleasant.
For me, the tourists who visit Huangshan is enjoyable.
For me, the tourists who visit Huangshan is funny.
For me, the tourists who visit Huangshan is positive.
